# Origins of JCHIMP

**DOI:** 10.3402/jchimp.v1i1.6328

**Published:** 2011-05-09

**Authors:** Robert P. Ferguson

With this, the first issue of the *Journal of Community Hospital Internal Medicine Perspectives* (www.jchimp.org), volume 1, the efforts of many individuals are no longer a concept but a reality. The journal's origins can be traced back to 2007 at the Association of Program Directors in Internal Medicine (APDIM) semi-annual meeting in Minneapolis, Minnesota. Prior to that meeting, the Department of Medicine at Union Memorial Hospital in Baltimore, Maryland had conducted a very successful research survey of internal medicine program directors in Pennsylvania, Maryland, and the District of Columbia. Using this regional success as a model, a national network was organized in Minneapolis. The informal Community Hospital Education and Research Network (CHERN) (www.cherninfo.com), an offshoot of the APDIM Community Hospital Assembly, was formed. Community hospitals sponsor more than half the Internal Medicine Residency Review Committee (RRC) accredited programs in the United States. Sponsoring internal medicine training requires substantial scholarly activity by residents and faculty as part of the RRC's scholarly requirements.^1^ CHERN was developed to serve as a vehicle for cooperative ventures in this area. Some community hospitals struggle to meet these research requirements because of institutional cultures and lack of resource support. Many community programs align themselves with local or statewide internal medicine organizations to provide a platform for scholarly activities. Statewide chapters of the American College of Physicians (ACP) are an excellent example. In Maryland, in 2010, there was 24 research and 149 clinical vignette oral presentations or posters presented by residents at the annual meeting. At my department in Union Memorial Hospital, we have taken it a step further and provided a summer research fellowship for rising second year medical students, mostly from the University of Maryland. These students, precepted by Union Memorial internal medicine faculty and working with medical residents, participate in research – often observational outcome studies. Although a number of manuscripts have been published, there is a very significant decline in publications compared with presentations. Part of this is due to the demise of the Maryland Medical Journal. Low subscription rates, high costs of paper journals, advertising issues, and very slow production turnaround have all played a part in this type of journal languishing.

Electronic journals have filled gaps worldwide. We are in the age of web-based e-Journals. Open access journals, based on the Internet and free to any reader with access to the Internet, continue to grow and can be accessed by clicking on a link on a home page. The page limits of paper journals and slow turnaround time do not exist here. David Solomon, from Michigan State University, a pioneer in the field, has written a commentary in this issue discussing the revolution of open access journals. On January 8, 2011, I accessed the Directory of Open Access Journals. At that time the directory listed 5,953 journals.^2^ By February 2011, this figure had grown to 6,085.^3^ A larger proportion of the titles listed are related to health and medicine, with a search on health specifically resulting in 417 journals. Of these, 246 journals were related to internal medicine, 68 of which were based in the United States. There were no journals that appeared to focus on community hospital settings or resident and student scholarship. There is a niche needing to be filled.

In the summer of 2010, during our 18th annual research fellowship, we began investigating the possibility of developing a community hospital based open access internal medicine journal. The objective was to develop an efficient electronic journal whose scope would include general internal medicine, particularly focusing on trainee research and on community hospitals’ perspectives. The journal was to be open access, online only and peer reviewed. Union Memorial Hospital Department of Medicine is the initial sponsor, and as the Chief of Medicine and Program Director I am the first editor. A voluntary editorial board of 15 was organized largely from Union Memorial faculty. We networked through the APDIM and at the Maryland ACP to develop a list of reviewers. As of April 2011, we had in excess of 100 reviewers and approximately 200 (and climbing) committed registered readers. We advertise locally, by word of mouth, and announcements. Initially, we expect a large proportion of articles to be invited. We have received commitments on 20 manuscripts that make up this issue and most of the next. We contracted with Co-Action Publishing in Stockholm to be our publisher.

Though the initial effort has largely been from one institution, our plan is for this to be a national journal with a diverse readership and editorial board, which may also be of interest to an international audience. Our 100 reviewers are drawn largely from APDIM members from around the country. Each issue will include general internal medicine in the sections of original research, brief reports, clinical vignettes, perspectives, commentaries, medical education, ECG images, and letters to the editor. A second type of issue will have themes, with invited papers.

We are now posting volume 1, issue 1 of the *Journal of Community Hospital Internal Medicine Perspectives*. In this issue, **David Solomon** describes open access medical publishing, where it has been, and what is its future. In an original research article, **Cheryl Quianzon**, a medicine fellow, reveals the prognostic value of an initial EKG in cocaine using patients admitted for chest pain. A medical education paper by **Rosalyn Stewart** reviews a new education model starting in year one for students in ambulatory offices in university and community settings, an excellent example of educational linkage between university and community sites. The JCHIMP ECG editor, **Marc Mugmon**, discusses an interesting ECG and clinical vignette and correlates ECG images with cardiac catheterization results. A resident case report by **Elena Forouhar** on urban leptospirosis points out once again that Baltimore alleys can be infectious places. The timeliness of the publication of the case report – 6 months from hospital presentation to Internet publication, points out the tremendous advantages of online journals.

We plan to publish quarterly initially. Our next issue will include invited articles on patient safety. Later this year, we intend to publish the Maryland ACP sponsored student and resident award winners from the 2011 meetings. Our journal is financially subsidized by the MedStar Institute for Innovation and the Union Memorial Hospital Department of Medicine. There is a $350 publishing fee to offset publication costs. No fees are incurred for invited articles or letters to the editor. All articles undergo a blind peer review by at least three reviewers in the field. There is no advertising in the journal. Except for publishing-associated costs and legal expenses, this is totally voluntary. We cannot guarantee a timeline at this point for publication, but we believe that our process should have a manuscript ready for publication a few months after it is submitted to JCHIMP. All reviews and communication are by email.

We would like to thank David Solomon. He is the architect who helped us make a structure out of a concept. We have leaned on him extensively to make this journal stand up and last. His support has been essential and we will continue to use his guidance in the future.

Finally, we invite all to participate in our journal as a reader or a reviewer. We encourage you to submit manuscripts on a variety of internal medicine clinical and educational issues, particularly those involving community hospitals, students, and residents.

*Robert P. Ferguson, MD* Editor

## Footnotes

1 Accreditation Council for Graduate Medical Education [Internet]. Chicago. ACGME. 2000-2011. Cited 2011 Feb 12. Program Requirements, Scholarly activities.

2 doaj.org[Internet] Directory of Open Access Journals. Lund University Libraries. 2011. Cited 2011 Jan 8. http://www.doaj.org

3 doaj.org[Internet] Directory of Open Access Journals. Lund University Libraries. 2011. cited 2011 Feb 2. http://www.doaj.org

